# Fabrication of Microfluidic Valves Using a Hydrogel Molding Method

**DOI:** 10.1038/srep13375

**Published:** 2015-08-24

**Authors:** Yusuke Sugiura, Hirotada Hirama, Toru Torii

**Affiliations:** 1 Department of Human and Engineered Environmental Studies, Graduate School of Frontier Sciences, The University of Tokyo, Kashiwa-shi, Chiba 277-8563, Japan

## Abstract

In this paper, a method for fabricating a microfluidic valve made of polydimethylsiloxane (PDMS) using a rapid prototyping method for microchannels through hydrogel cast molding is discussed. Currently, the valves in microchannels play an important role in various microfluidic devices. The technology to prototype microfluidic valves rapidly is actively being developed. For the rapid prototyping of PDMS microchannels, a method that uses a hydrogel as the casting mold has been recently developed. This technique can be used to prepare a three-dimensional structure through simple and uncomplicated methods. In this study, we were able to fabricate microfluidic valves easily using this rapid prototyping method that utilizes hydrogel cast molding. In addition, we confirmed that the valve displacement could be predicted within a range of constant pressures. Moreover, because microfluidic valves fabricated using this method can be directly observed from a cross-sectional direction, we anticipate that this technology will significantly contribute to clarifying fluid behavior and other phenomena in microchannels and microfluidic valves with complex structures.

Pneumatic valves in polydimethylsiloxane (PDMS) microchannels are used in various applications as control mechanisms for sample transportations and to facilitate mixing^1^,^2^,^3^,^4^,^5^,^6^. The valve mechanism within a microchannel is formed by a structure in which a PDMS thin film is interposed between two microchannels. These channels are differentiated into a control channel and a main channel. When pneumatic pressure applied to the control channel is changed, the PDMS thin film is deformed, and the flow in the main channel can be controlled. PDMS is easily deformed by pneumatic pressure; however, the mechanical properties of PDMS do not change, even if the material is repeatedly deformed^7^,^8^. Therefore, a valve mechanism installed in a microchannel cannot only control complex flow^2^,^9^,^10^, it can also function as a fluid gage or pump mechanism in a microchannel^3^,^11^,^12^. Devices that use valves to implement protein crystallization^13^,^14^ or DNA integrated analysis^15^,^16^,^17^ are being developed, and valve mechanisms will likely become an even more critical element in microchannels in the future.

In conjunction with the expansion of microfluidic devices, various techniques are being proposed that can be used to prototype microchannels rapidly. Photolithography is currently the most widely used technology for rapid prototyping of microchannels. In photolithography, a resist pattern fabricated using such materials as ultraviolet curable resins are transferred to fabricate a microchannel. In addition, the application of microchannels fabricated through hot embossing or cutting is under development. With these techniques for the rapid prototyping of microchannels, high fabrication precision and mass production can be easily realized. However, these techniques are limited in their ability to fabricate microchannels with three-dimensional structures^18^. Furthermore, when microchannels are fabricated using conventional techniques, it is difficult to observe them from a cross-sectional direction or any other free direction. The hydrogel molding method (HGM method) enables the rapid prototyping of microchannels without the use of photolithography^19^. One feature of the HGM method is that it does not require special equipment in the fabrication process; fabrication can be achieved using only typical equipment found in laboratories. Furthermore, with the use of conventional techniques, the fabrication of microchannels with complex cross-sectional shapes or three-dimensional structures requires a series of complex steps. However, the HGM method has enabled easy fabrication and application of complex structures^20^.

The drive principle of valve mechanisms through pneumatic pressure was clarified through analysis using the finite element method^21^. However, advanced techniques were required to observe valves driven in microchannels^22^,^23^ because when microchannels are fabricated using a conventional technique with a three-dimensional structure, it is difficult to observe them from a cross-sectional direction.

In this study, we used the ability to fabricate a stereoscopic microchannel structure easily using the HGM method to create a microfluidic valve that can be observed not only from the layered direction of the three-dimensional structure but also from the cross-sectional direction. Next, we examined the fabricated microfluidic valve.

## Methods

In the microfluidic valve, the channel was opened and closed by deforming the PDMS thin film. The displacement, *d,* of the PDMS thin film is determined by the physical properties and structure of the film, as well as by the pressure, *P,* applied to the film. This pressure can be expressed by the following equation^21^:


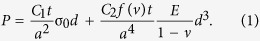


**Figure 1:**
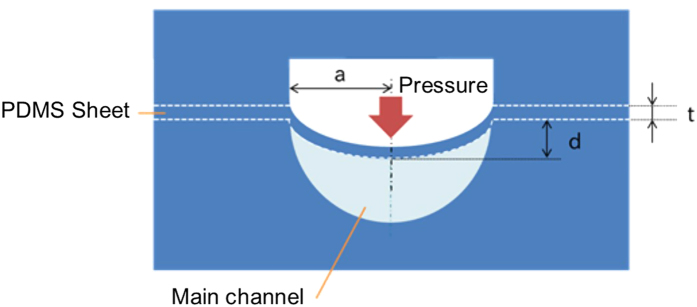
Schematic of the valve mechanism.

Similar to Pan *et al.*^21^, *C*_*1*_, *C*_*2*_, and *f(ν)* are determined by the shape of the valve structure; *a* and *t* express the dimensions of the valve thin film (Fig. 1); and σ_0_ (residual stress), *ν* (Poisson’s ratio), and *E* (Young’s modulus) express the physical properties of the PDMS. A certain hydraulic pressure, the Laplace pressure, is required in the fluid that is transported to the main microchannel to pass under the thin film in the valve mechanism. The Laplace pressure, *Δp*, is determined by the shape of the channel (hydraulic diameter), *D*_*h*_, the interfacial tension, *γ*, of the flowing fluid, and the contact angle, *θ* and is expressed as follows:


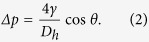


On the basis of this equation, the relationship between the pneumatic pressure applied to the valve and the Laplace pressure of the valve channel can be determined.

**Figure 2:**
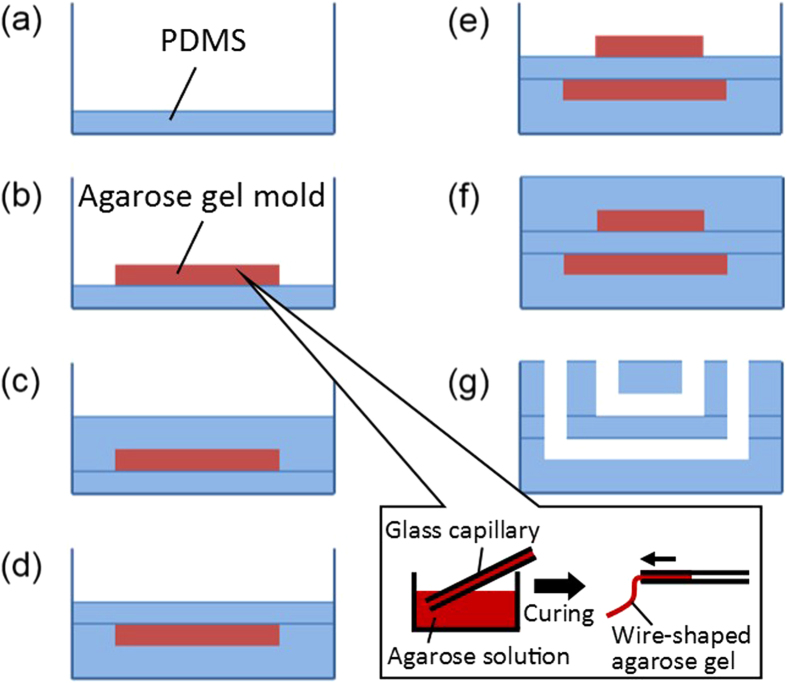
Procedures for fabricating a valve channel: (**a**) the PDMS thin film is spread out, (**b**) an agarose gel mold (which was prepared by filling agarose solution into a glass capillary, curing it at 4 °C, and ejecting a cured wire-shaped agarose gel from the capillary) is placed on top of the thin film, and (**c**) uncured PDMS is poured into the mold. The PDMS is then heated for 2 h in a 50 °C oven, and then (**d**) the PDMS is removed, turned upside down, and positioned back in the container. (**e**) The agarose gel mold is arranged on the opposite surface of the thin film, and (**f**) the uncured PDMS is poured into the mold. The PDMS is again heated for 2 h in a 50 °C oven and later for 30 min in a 75 °C oven. (**g**) Next, holes are drilled in the PDMS, and the agarose gel is flushed out using hot water to complete the PDMS valve channel. When the fixture shown in Fig. 3d is installed, in procedure (**d**), the PDMS is cut with a knife such that the PDMS can fit into the fixture.

The procedures for fabricating a microchannel with a valve using the HGM method^19^,^20^ are presented in Fig. 2. A three-dimensional structure can be fabricated by repeating hydrogel cast molding and PDMS layering. Observation from a cross-sectional direction is enabled using a fixture in the microchannel process. For the structure of the valve channel, the control channel for pneumatic pressure application was fabricated using agarose gel prepared using a glass tube (1 mm × 1 mm, square cross-section) as the casting mold, and the main channel through which the aqueous solution is transported was fabricated using agarose gel prepared using a glass tube (ϕ 1 mm, θ-shaped cross- section, Theta Tubing^24^) as the casting mold. The fabricated microfluidic valve had a structure in which PDMS thin film was interposed between the control channel and the main channel, and the microchannels opened and closed by the deformation of the square-shaped valve thin film.

## Results

**Figure 3:**
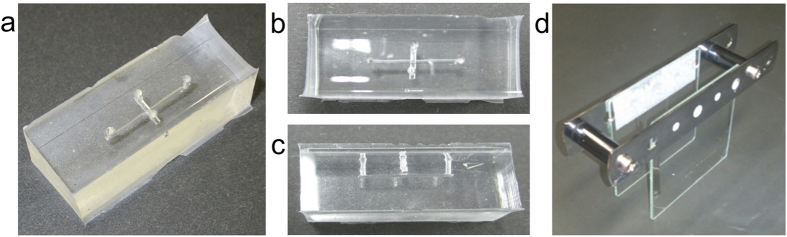
A microfluidic valve for observation fabricated using the HGM method. (**a**) External view of the microchannels. (**b**) View from a layered direction. (**c**) View from a cross-sectional direction. (**d**) Fixture used when fabricating the microchannels.

Figure 3 presents photographs of a fabricated microfluidic valve. The fabrication of the three-dimensional structure using the HGM method was expanded to construct a valve structure. The fabricated PDMS microchannel could be observed from both the layered and the cross-sectional directions. A fixture (Fig. 3d) was used for fabricating the PDMS channel, and the PDMS was cured to obtain a flat surface on the side of the channel. When the PDMS was cut with a knife after curing, the cutting surface became rough, which made observation from the side surface difficult.

**Figure 4:**
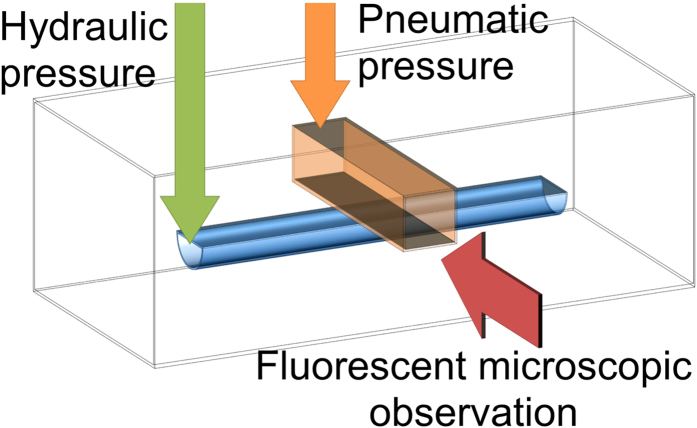
Schematic observational view of a microfluidic valve. Opening and closing of the valve is controlled by applying pneumatic pressure to the control channel, which has a square cross-section, and hydraulic pressure to the main channel, which has a semicircular cross-section. Opening and closing of the valve is observed from fluorescence microscopy images captured from the cross-sectional directions.

The opening and closing of the valve in the microchannel was observed using a fluorescence microscope (Fig. 4). The microchannel for observation was configured using a control channel and a main channel, and the flow of aqueous solution in the main channel was hindered by applying pneumatic pressure to the control channel. To confirm the opening and closing of the valve, a fluorescent aqueous solution (fluorescein sodium) was introduced into the main channel through a Teflon tube (outer diameter: 1.6 mm, inner diameter: 0.5 mm). For the experimental conditions, the microchannel was prepared using two types of valves with thicknesses of 100 μm and 70 μm, and the pneumatic pressure applied to the valve was controlled in the range from 0 to 200 kPa with respect to the gage pressure. The hydraulic pressure of the aqueous solution was increased in steps of 50 Pa, and the aqueous solution was observed at each pneumatic pressure level until it began flowing through the valve.

**Figure 5:**
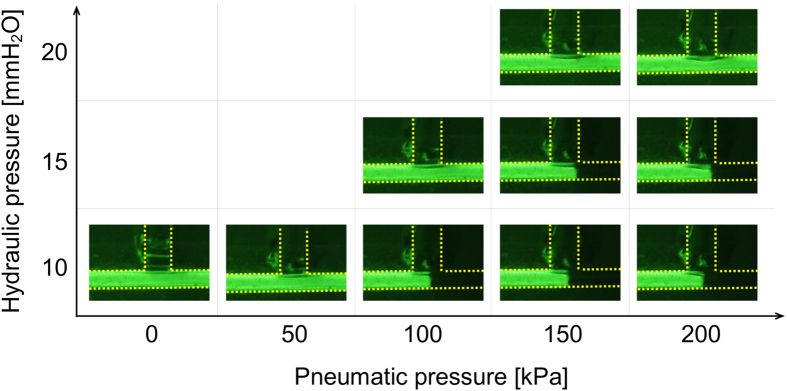
Results for observations of valve opening and closing with respect to valve control pneumatic pressure and microchannel hydraulic pressure. In each image, the pneumatic pressure is applied from the top and the hydraulic pressure is applied from the left. The thickness of the PDMS thin film of the valve mechanism is 100 μm, and the deformation occurs at the intersection shown in the images.

We confirmed that the microchannel could be opened and closed by controlling the pneumatic pressure applied to the valve (Fig. 5). The microfluidic valve operation conditions were observed from fluorescence microscopy images captured from the valve cross-sectional direction, which is difficult in a conventional microchannel fabrication technique with a three-dimensional structure. The amount of deformation of the PDMS thin film in the valve mechanism was also observed to change with the change in the applied pneumatic pressure. In conjunction with this change, the amount of hydraulic pressure required to pass through the valve mechanism also changes.

**Figure 6:**
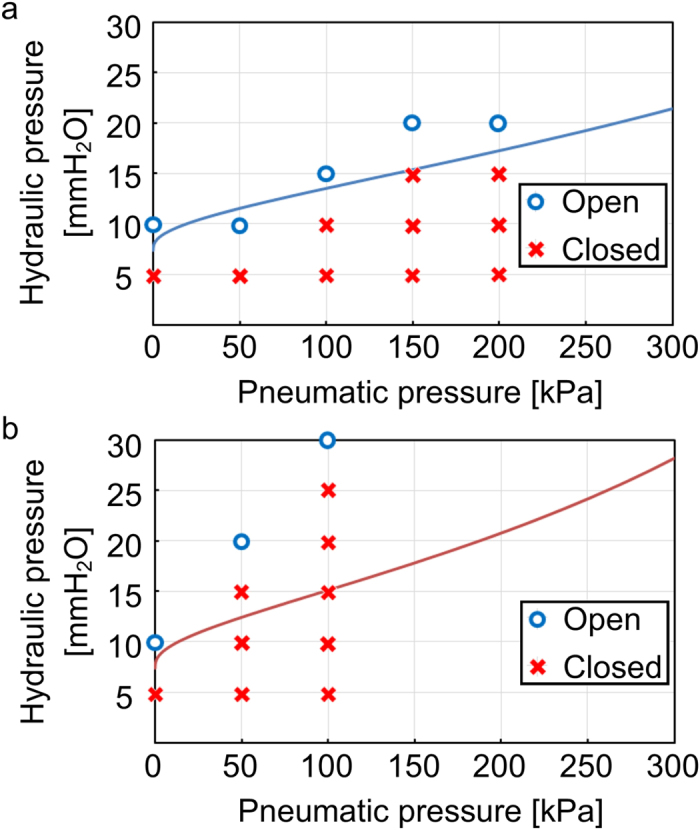
Results from observing the opening and closing of the microfluidic valve. (**a**) PDMS thin film with a thickness of 100 μm and (**b**) PDMS thin film with a thickness of 70 μm. The solid line in each graph shows the calculated theoretical value for the opening/closing boundary.

It was also confirmed that the conditions to open and close the microfluidic valve are related to the pneumatic pressure and that these conditions are significantly affected by the thickness of the thin film (Fig. 6). As the valve pneumatic pressure is increased, the channel exhibits a tendency to close more strongly than the theoretical value. From these results, the theoretical value is assumed to be valid in a range in which the thin-film deformation is small. Moreover, when a strong pneumatic pressure was applied to the valve, we observed that air permeated from the PDMS thin film and that air bubbles were generated in the aqueous solution. Air bubbles in the aqueous solution cause a larger resistance to the flow of the solution than the valve; therefore, time observations were performed within a range of pneumatic pressure that does not generate air bubbles.

## Discussion

When the theoretical calculations and actual observation results were compared, we observed that for the film thickness of 100 μm, the results were close to the predicted results. However, when the film thickness was 70 μm, the channel was blocked more easily than predicted. The hydrophilic property of the surface of the PDMS in the microchannel fabricated might be altered using the HGM method compared to an ordinary PDMS surface.

In regards to the generation of air bubbles in the microfluidic valve, the generation of pneumatic pressure resulted in the formation of air bubbles when the PDMS film was thin, and the pneumatic pressure was relatively high. This phenomenon is observed possibly because of air leakage from the control channel to the main channel through the thin film configuring the valve mechanism due to the air permeability of the PDMS^25^. At the time of fabrication, the PDMS contains a certain amount of air bubbles. As the PDMS film is made thinner, very thin areas are generated. Moreover, PDMS has a high level of air permeability and can easily leak air. Therefore, controlling the air permeability of the PDMS thin film in the valve mechanism by surface treating the PDMS is necessary^26^,^27^.

In conclusion, we developed a rapid prototyping method for HGM-based fabrication of a microchannel containing a PDMS thin-film valve structure and confirmed the operation of the valve structure. Consequently, we confirmed that the valve displacement could be predicted within a range of constant pressures. As the result, it was confirmed that the microchannel fabricated with the hydrogel molding method acted as a microfluidic valve. This fabrication method has the following advantages. (1) Rapid prototyping using the HGM method, which can be used to fabricate a microchannel with a hemisphere cross-section, is effective for fabrication of a microfluidic valve because a microchannel with a hemisphere cross-section, rather than square one, is needed for closing a microfluidic valve^28^. (2) The cost of this method is lower than photo- and soft-lithography^19^. (3) By using an automatic gel-drawing method, such as 3D gel printing^29^, a microfluidic valve with a diverse shape could be automatically and precisely fabricated. (4) A fabricated microfluidic valve enables direct observation of valve displacement. This development is not only useful as a technique for evaluating valve designs but has also demonstrated that three-dimensional structures in microchannels can be observed from a free direction. It is expected that this development will help to elucidate various phenomena in microchannels.

## Additional Information

**How to cite this article**: Sugiura, Y. *et al.* Fabrication of Microfluidic Valves Using a Hydrogel Molding Method. *Sci. Rep.* **5**, 13375; doi: 10.1038/srep13375 (2015).
